# Do Circulating Extracellular Vesicles Strictly Reflect Bronchoalveolar Lavage Extracellular Vesicles in COPD?

**DOI:** 10.3390/ijms24032966

**Published:** 2023-02-03

**Authors:** Mariaenrica Tinè, Tommaso Neri, Davide Biondini, Nicol Bernardinello, Alvise Casara, Maria Conti, Marianna Minniti, Manuel G. Cosio, Marina Saetta, Alessandro Celi, Dario Nieri, Erica Bazzan

**Affiliations:** 1 Department of Cardiac, Thoracic, Vascular Sciences and Public Health, University of Padova, 35128 Padova, Italy; 2 Centro Dipartimentale di Biologia Cellulare Cardiorespiratoria, Dipartimento di Patologia Chirurgica, Medica, Molecolare e dell’Area Critica, Università degli Studi di Pisa, 56124 Pisa, Italy; 3 Meakins-Christie Laboratories, Respiratory Division, McGill University, Montreal, QC H3A 0G4, Canada

**Keywords:** COPD, extracellular vesicles, bronchoalveolar lavage

## Abstract

Cell-derived extracellular vesicles (EVs) found in the circulation and body fluids contain biomolecules that could be used as biomarkers for lung and other diseases. EVs from bronchoalveolar lavage (BAL) might be more informative of lung abnormalities than EVs from blood, where information might be diluted. To compare EVs’ characteristics in BAL and blood in smokers with and without COPD. Same-day BAL and blood samples were obtained in 9 nonsmokers (NS), 11 smokers w/o COPD (S), and 9 with COPD (SCOPD) (FEV1: 59 ± 3% pred). After differential centrifugation, EVs (200–500 nm diameter) were identified by flow cytometry and labeled with cell-type specific antigens: CD14 for macrophage-derived EVs, CD326 for epithelial-derived EVs, CD146 for endothelial-derived EVs, and CD62E for activated-endothelial-derived EVs. In BAL, CD14-EVs were increased in S compared to NS [384 (56–567) vs. 172 (115–282) events/μL; *p* = 0.007] and further increased in SCOPD [619 (224–888)] compared to both S (*p* = 0.04) and NS (*p* < 0.001). CD326-EVs were increased in S [760 (48–2856) events/μL, *p* < 0.001] and in SCOPD [1055 (194–11,491), *p* < 0.001] when compared to NS [15 (0–68)]. CD146-EVs and CD62E-EVs were similar in the three groups. In BAL, significant differences in macrophage and epithelial-derived EVs can be clearly detected between NS, S and SCOPD, while these differences were not found in plasma. This suggests that BAL is a better medium than blood to study EVs in lung diseases.

## 1. Introduction

Extracellular vesicles (EVs) are membrane particles released by cells. The term includes exosomes, microvesicles, and other vesicular components [1,2,3,4,5]. EVs are abundantly present in extracellular fluids, including circulating blood, cerebrospinal fluid, and urine, and carry a variety of proteins, nucleic acids, lipids, and metabolites as their cargoes. Among other functions of EVs, intercellular transfer of their cargoes from donor to recipient cells enables non-cell autonomous control of the immune response, gene transcription, and other cellular functions in multicellular organisms [1,2,3,4,5].

EVs have been extensively studied as potential biomarkers. Blood is one of the most promising sources for EV-related biomarker development, owing to the ease of sampling with minimal invasion. To date, numerous studies of biomarker candidates from blood-derived EVs have been reported for different types of cancers [6,7,8], immune diseases [9,10], and neurodegenerative disorders, such as Alzheimer’s and Parkinson’s disease [11,12]. However, the development of reliable EV-based biomarkers suitable for clinical use remains challenging, as even within the same disease the results of EVs analyses of patients’ blood are often inconsistent among studies [13,14,15]. A major reason for these inconsistent results is the difference in experimental conditions because blood EVs profiles are likely to be affected by various experimental factors, such as the isolation method and the choice of the sample source (plasma or serum). Furthermore, an even higher level of complexity is expected in the study of human samples, where the interferences of coexisting conditions are unpredictable. A fitting example of such a challenge is the study of chronic obstructive pulmonary disease (COPD), a respiratory condition characterized by persistent inflammation of the airways that, in susceptible smokers, is sustained by the host immune response [16]. Several studies have investigated circulating EVs in COPD patients, showing an increased amount of endothelial cell-derived EVs [17,18,19], particularly during exacerbations [20,21]. Nonetheless, increased peripheral levels of blood cell-derived EVs are often described in vascular disorders as well, such as stroke [22,23] and venous thromboembolism [24], conditions that can coexist with COPD [25]. Consequently, it is often difficult to discern the actual contribution of COPD itself from that of its systemic inflammatory extrapulmonary manifestations in circulating EV levels. To overcome this potential limitation, we [26] and others [27,28] have focused on the study of samples directly derived from the airways, rather than blood, in COPD. However, to what extent circulating EVs reliably mirror airway EVs in COPD is an issue that has never been directly approached. The goal of this study was to provide evidence of the possible similarities and differences between EVs detected in blood and those identified in bronchoalveolar lavage (BAL) in COPD.

## 2. Results

The subjects’ clinical characteristics are shown in Table 1. Smokers with COPD and without COPD were older than nonsmokers (*p* < 0.05).

To analyze whether BAL and blood are comparable sources of EVs, we prepared and isolated BAL and blood EVs by conventional differential centrifugation methods. In 85% of the cases, the dimensions of the EVs ranged between 200 and 500 nm (a range that excludes apoptotic bodies), and there was no difference between the three groups regarding EVs size (*p* = 0.51).

**Figure 1:**
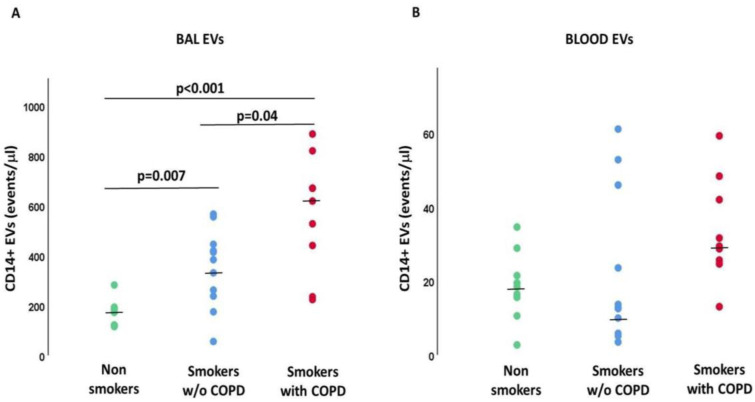
Macrophage-derived EVs (CD14+ events/μL) in BAL (**A**) and Blood (**B**). *p* values in the figure represent the results of Mann-Whitney U tests. Kruskal-Wallis test: *p* < 0.001 for BAL EVs. Kruskal-Wallis test: *p* = NS for Blood EVs.

The number of EVs/μL derived from BAL macrophages (CD14+) was significantly higher in smokers with (*p* < 0.001) and without COPD (*p* = 0.007) than in nonsmokers and also significantly higher in smokers with COPD than in smokers without COPD (*p* = 0.046, Figure 1A). By contrast, the number of EVs/μL derived from plasma macrophages was not significantly different between the three groups of subjects analyzed (Figure 1B). Of note, the number of macrophage-derived EVs detected in plasma was significantly lower than those of BAL (*p* < 0.001).

**Figure 2:**
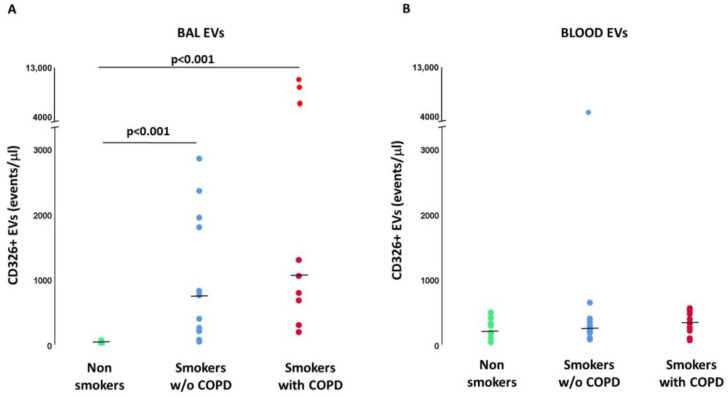
Epithelial-derived EVs (CD326+ events/μL) in BAL (**A**) and Blood (**B**). *p* values in the figure represent the results of Mann-Whitney U tests. Kruskal-Wallis test: *p* < 0.001 for BAL EVs. Kruskal-Wallis test: *p* = NS for Blood EVs.

The number of EVs/μL derived from BAL epithelial cells (CD326+) was significantly higher in smokers with (*p* < 0.001) and without COPD (*p* < 0.001) than in nonsmokers (Figure 2A). In contrast, the number of EVs/μL derived from plasma epithelial cells was not significantly different between the three groups of subjects (Figure 2B).

**Figure 3:**
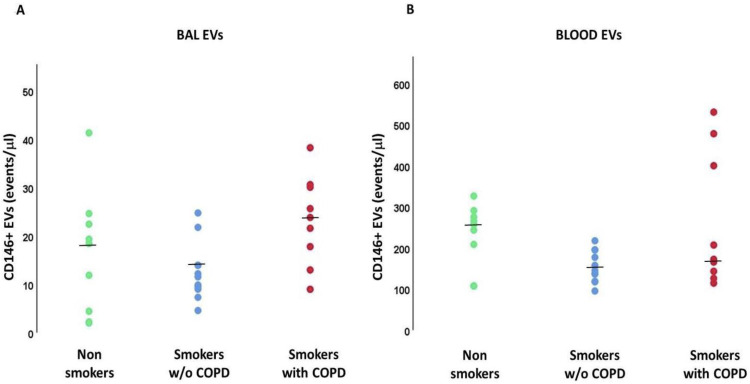
Endothelial-derived EVs (CD146+ events/μL) in BAL (**A**) and Blood (**B**). *p* values in the figure represent the results of Mann-Whitney U tests. Kruskal-Wallis test: *p* < 0.001 for BAL EVs. Kruskal-Wallis test: *p* = NS for Blood EVs.

The number of EVs/μL derived from BAL total endothelial cells (CD146+) was not significantly different among the three groups analyzed (Figure 3A), as well as in plasmatic endothelium-derived EVs (Figure 3B). Of note, the number of endothelium-derived EVs detected in BAL was significantly lower than that in plasma (*p* < 0.001).

**Figure 4:**
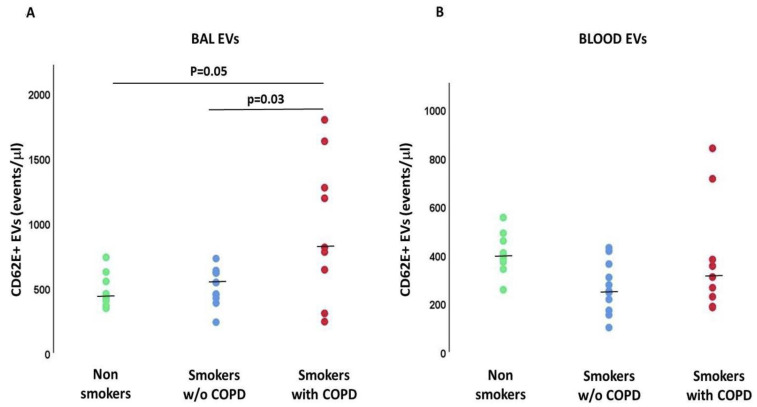
Endothelial activated-derived EVs (CD62E+ events/μL) in BAL (**A**) and Blood (**B**). *p* values in the figure represent the results of Mann-Whitney U tests. Kruskal-Wallis test: *p* < 0.001 for BAL EVs. Kruskal-Wallis test: *p* = NS for Blood EVs.

The number of EVs/μL derived from activated endothelial cells (CD62E+) in BAL was significantly higher in smokers with COPD than in smokers without COPD (*p* = 0.03) and nonsmokers (*p* = 0.05) (Figure 4A). In contrast, the number of EVs/μL derived from activated endothelial cells in plasma did not differ among the three groups of subjects analyzed (Figure 4B). Of note, the number of activated endothelial cell-derived EVs detected in BAL was significantly higher than those in plasma (*p* < 0.001).

Of note, comparing current and ex-smokers, no difference in the different types of EVs was observed between the two groups. 

**Figure 5:**
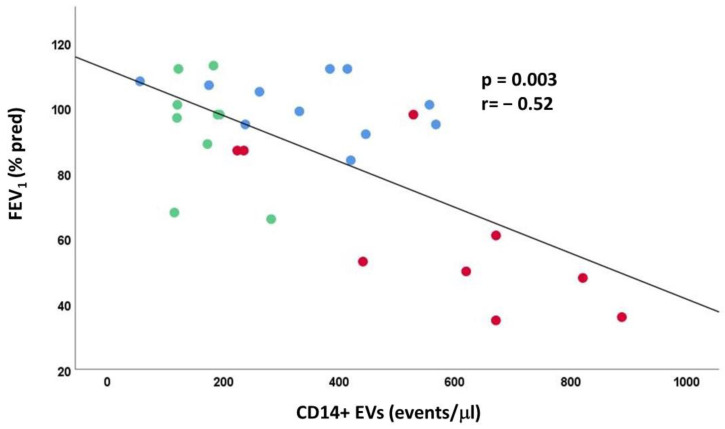
Correlation of lung function (FEV1 pred%) with macrophages derived-EVs. Red circles: Smokers with COPD; Lightly blue circles: Smokers without COPD; Green circles: Non-smokers. Spearman rank correlation (r = −0.52 and *p* = 0.003).

When all subjects were analyzed together, the macrophage-EVs, epithelial-EVs, and endothelial-EVs in BAL were inversely correlated with lung function parameters, like FEV_1_ % pred [r = −0.52, *p* = 0.003 (Figure 5); r = −0.35, *p* = 0.05; r = −0.52, *p* = 0.01 respectively] and FEV_1_/FVC % [r = −0.73, *p* < 0.001; r = −0.53, *p* = 0.003; r = −0.38, *p* = 0.04 respectively). 

When all smokers (current and ex-smokers) were analyzed together, macrophage-EVs and endothelial-EVs in BAL were inversely correlated with FEV_1_ % pred (r = −0.64, *p* = 0.002; r = −0.55, *p* = 0.01 respectively) and FEV_1_/FVC % (r = −0.64, *p* = 0.002; r = −0.44, *p* = 0.05 respectively).

The correlation between FEV_1_ % pred and macrophage-EVs remained significant when only subjects with COPD were considered (r = −0.69, *p* = 0.04). 

Conversely, no correlation was found between blood EVs and functional parameters (FEV1% predicted and FEV_1_/FVC%).

## 3. Discussion

Although circulating EVs have been extensively studied in COPD [17,18,19,20,21,29], the coexistence of systemic inflammation and smoking-related diseases potentially undermines their specificity as biomarkers in these patients. Airway-derived EVs have the potential to overcome this limitation. EVs were first identified in the BAL of healthy subjects in 2003 [30]. Afterward, several studies investigated EVs within airway secretions, on the assumption that they might reliably mirror the inflammation characterizing obstructive lung diseases. Specific EVs were isolated in sputum samples of cystic fibrosis patients [31], nasal lavage of asthmatics [32], and BAL of COPD patients [26,27,33]. 

Our study of EVs in both blood and BAL from smokers with and without COPD and non-smoking controls showed deep discrepancies between the two studied sources. Bronchoalveolar lavage (BAL) fluid was significantly enriched in macrophage-(CD14+), epithelial-(CD326+), and activated endothelial cell-derived EVs (CD62E+) as compared to blood samples. The expression of CD62E, a marker of activated endothelial cells expressing E-selectin, which is crucial for inflammatory cells recruitment and accumulation in the sites of inflammation [34,35], was higher in the BAL of smokers with COPD compared to control smokers and nonsmokers, a trend not reflected by plasma assessment. Conversely, non-activated endothelial cell-derived EVs (CD146+ a marker for cell-cell adhesion and permeability [36]) were much more abundant in plasma than BAL but were similar in all groups studied, regardless of the source. Since CD62E is synthesized after activation by inflammatory stimuli [34,35], the higher values of CD62E+ EVs in BAL of COPD patients with respect to both smokers without COPD and nonsmokers could express significant inflammatory damage of pulmonary vascular endothelium, pathologically related to COPD itself, and not completely explained by the smoking history. Both epithelium- and macrophage-derived EVs were significantly increased in BAL of smokers with and without COPD compared to nonsmokers. Epithelium-derived EVs did not differ between smokers with and without COPD, suggesting that their increase is due to cigarette smoke exposure rather than to the disease itself. Preclinical studies have shown that cigarette smoke exposure can increase EV secretion from human airway epithelial cells [37,38] and modify their content [39]. These cigarette smoke-induced EVs can by themselves be harmful, promoting the shift of alveolar macrophages obtained from patients with familial emphysema towards a pro-inflammatory phenotype [40] and sharing procoagulant properties that may contribute to the increased cardiovascular and respiratory risk observed in smokers [41]. A robust increase in epithelial cell-derived EVs was observed as a consequence of hyperoxia-associated oxidative stress in mice BAL, suggesting that EVs shedding from airway cells might represent a common reaction to noxious stimuli [42,43].

In our study, COPD patients had the highest amount of macrophage-derived EVs in BAL compared to both healthy smokers and nonsmokers. Moreover, CD14+ EVs correlated with lung function impairment (expressed by FEV1% predicted) and with the presence of airflow obstruction (expressed by FEV1/FVC%), thus confirming our previous results [26]. Interestingly, the significant relationship between CD14+ EVs and FEV1% predicted in COPD patients suggested a role for these EVs as biomarkers of severity in COPD. Macrophages have long been recognized in COPD pathogenesis [44]: in COPD, the alveolus milieu depends on the interaction between epithelial cells and alveolar macrophages, one of the barriers against inhaled noxious agents, including smoke, pollutants, and infectious agents [45]. EVs are thought to mediate this cross-talk, becoming crucial in both homeostasis maintenance and inflammation development [45]. In this light, the relationships we found between CD14+ EVs and FEV1 in all smokers (both with and without COPD) is consistent with a role for these EVs in the early events characterizing the development of the disease. A previous study has already demonstrated that plasmatic endothelium-derived EVs were potential biomarkers of early lung destruction before overt COPD (i.e., subjects with markers of emphysema and without airflow obstruction) [46], but, to our knowledge, no studies have compared blood-borne and BAL-derived EVs so far. Noteworthily, the current literature in the field has recently suggested the term “pre-COPD”, referring to these early stages of disease development [47]. Whether CD14+ EV might represent a suitable marker for the detection of pre-COPD remains to be elucidated.

Recently, alveolar macrophage-derived EVs (CD11c−F4/80+ events) have been described in murine BAL, where they represent the main source of microvesicles [42]. Soni et al. performed a comprehensive analysis of EVs in BAL and blood samples of patients with COPD. They looked for leukocyte, epithelial, and endothelial cell-derived EVs and found a significant increase in neutrophil-derived microvesicles in COPD patients compared to healthy volunteers. In this study, monocyte/macrophage-derived EVs were not increased in COPD patients as compared to healthy individuals, a result that might be linked to the different sorting strategies in FACS and to the lack of a control smoker group [27]. Of note, in parallel with our results, they found significant signals (TNF, IL-1b, IL6, and CXCL8) from the study of EVs in the BAL, while the plasma assay revealed similar EV populations amongst COPD patients and healthy controls.

It is well known that COPD is characterized by significant airway inflammation [16], that can ultimately spill over into the systemic blood circulation and be responsible for the so-called extrapulmonary manifestations of the disease [48]. Based on these observations, one could expect to find similar EV patterns in BAL and in the blood. The different profiles we have found in EV populations (and in their relative proportions) between BAL and blood can thus reflect the activation of different inflammatory pathways in COPD, suggesting that the simple spillover of inflammatory mediators from airways into the blood represents an unsatisfactory explanation of the complex pathogenic mechanisms of the disease. Indeed, since COPD is a complex and heterogeneous disease, identifying specific mechanisms of the disease (called “endotypes”) and their corresponding biomarkers is of utmost importance for the appropriate management of this disabling disease [49]. In this light, it is conceivable that EVs in BAL could better reflect the airway inflammation, which is definitely associated with COPD development and progression, thus representing an attractive biomarker for this process.

Indirect signals of the higher reliability of body fluids in direct contact with the disease’s primary site as compared to circulating mediators (including EVs) are available. In the field of COPD, a different profile in other biomarkers (like inflammatory cells) between blood and lung tissue has been described. For example, there is no direct relationship between circulating and lung tissue eosinophils [50]. Similarly, in patients with prostate cancer, the EVs in urinary and serum supernatant were dissimilar and only urinary EVs markers were clearly associated with cancer aggressiveness [6]. The proteomic fingerprint of the EVs in the central nervous system, enclosed from peripheral circulation by hematoencephalic barrier, is preserved in cerebrospinal fluid and tears, providing a base for the study of multiple sclerosis [10]. Focusing on BAL, it offers a better sample for genotyping in non-small cell lung cancer when compared to blood [51]. Our study highlights, as a direct objective, the discrepancies between airway and plasma EVs in COPD and supports the reliability of BAL for the study of EVs in disease development and for the identification of biomarkers. Alveolar macrophage-derived EVs, especially, might convey crucial information on COPD pathogenesis and emphysema progression and, in feature, reveal therapeutic targets. Of course, further studies are needed to understand the real potential of airways-borne EVs, isolated in the BAL, as players in COPD pathogenesis (including the extrapulmonary manifestations).

Our study has methodological limitations. First, EV types were analyzed using FACS analysis only, and many other techniques could be applied to complement this approach and further expand our understanding of airway EV secretion as disease biomarkers. We focused only on epithelial, endothelial, and macrophage-derived EVs and investigation of other leukocyte-derived EVs will be addressed in the future. Finally, an important limitation is the low power of the study due to the small number of subjects in each group. However, despite the small number of patients, we were able to detect changes in several subpopulations of EVs that are consistent with our previously described findings in bronchoalveolar lavage, and we believe that our study provides preliminary data that may prove useful for designing future research in this field.

We can conclude that BAL fluid offers a more powerful source for the study of EVs in COPD compared to blood where signals are blurred by coexisting morbidities, typically occurring in smokers. It is desirable that future efforts will be focused on the characterization of EV populations in airway secretions to discover novel therapeutic targets and reliable biomarkers of COPD.

## 4. Materials and Methods

### 4.1. Clinical Characteristics

BAL and blood samples were obtained according to standard protocols (7) in 9 nonsmokers [forced expiratory volume in 1 s (FEV1): 101 ± 4% predicted], 11 smokers without COPD (FEV1: 93 ± 8% predicted), and 9 smokers with COPD (FEV1: 59 ± 3% predicted) undergoing clinically indicated bronchoscopy (i.e., assessment of suspect lung cancer: in these cases, BAL was always performed in the lung opposite to the lesion). Of the 20 smoker patients, 8 were current smokers, and 12 were ex-smokers who had quit smoking a minimum of 1 year before entering the study. Spirometry was performed according to international guidelines, and smokers with fixed airflow limitation—defined as ratio of forced expiratory volume in one second (FEV1) to forced vital capacity (FVC) of <0.7 post-bronchodilation—and consistent clinical history were defined as COPD, according to the recent GOLD 2023 document [25]. Smokers without fixed airflow limitation—defined as ratio of forced expiratory volume in one second (FEV1) to forced vital capacity (FVC) of >0.7 post-bronchodilation—and without any other clinical, functional, or radiologic signs suggestive of chronic lung abnormalities were defined as smokers without COPD, exhibiting no evidence of underlying lung disease. Finally, non-smokers had spirometry with FEV1/FVC > 0.7 and were never smokers (≤1 pack-year history of tobacco smoking).

During the month preceding the study, patients with COPD had no exacerbations, defined as increased dyspnoea associated with a change in the quality and quantity of sputum, leading the subject to seek medical attention. All subjects were clinically stable and free of major comorbidities. Subjects with asthma or history of asthma, a1-antitrypsin deficiency, bronchiectasis, autoimmune diseases, other respiratory diseases, infections, cardiovascular diseases, and those on systemic or inhaled corticosteroids were excluded from the study. The study was approved by Ethics Committee (Ref. No. 0006045), and all subjects gave written informed consent before their enrollment.

### 4.2. EV Isolation

BAL and blood fluids were obtained [2,26] and immediately processed. 

BAL samples were filtered by a gauze filter (50-µm-size pore) to remove any mucus and centrifuged at 350 g for 10 min at room temperature, to separate supernatant from BAL cells. The BAL supernatants were centrifuged at 10,000× *g* for 30 min at 4 °C to isolate EV pellets. Finally, EVs were resuspended in ultrafiltered PBS and stored at 80 °C [26,29].

Blood (4 mL) was drawn into sodium citrate. Platelet-poor plasma (PPP) was obtained by two subsequent centrifugations: 1500× *g* for 15 min and 13,000× *g* for 2 min at room temperature. PPP was stored at −80 °C.

### 4.3. EV Characterization

The pre-analytic phase of EV analysis has previously been reported [26]. Flow cytometry analysis was performed using a CytoFLEX flow cytometer (Beckman Coulter, Brera, CA, USA), as previously reported [26]. For EV size calibration of the flow cytometer, fluorescent polystyrene beads (Megamix FSC & SSC Plus, BioCytex, Marseille, France) were used in sizes of 0.1, 0.16, 0.2, 0.24, 0.3, 0.5, and 0.9 μm. Violet side scatter (VSSC) and FL1 channel gain were set to visualize the beads. The side scatter (SSC) from the 405 nm violet laser (VSSC) was used as a trigger signal to discriminate the noise. Megamix bead solution was gated, excluding the background noise (because of the solution itself). After turning the set in VSSC and forward scatter (FSC), a rectangular gate was set between the 0.1 μm and 0.9 μm bead to select particles that might be included in the range of exosomes and ectosomes and exclude larger vesicles such as apoptotic bodies, usually falling in the 1–4 μm range of size.

For the characterization and analysis of the EVs, 20 μL of samples were stained with 10 μL of calcein-AM (Sigma-Aldrich, Milan, Italy), to confirm the presence and integrity of EVs. Samples were then incubated in the dark for 30 min at room temperature with 2 µL of fluorescent-conjugated monoclonal antibodies against cell-type specific antigens. Stained samples were then diluted by adding 140 μL of sterile filtered PBS. 

Macrophage-derived EVs were identified using CD14-APC (allophycocyanin, eBioscience, San Diego, CA, USA). Epithelial-derived EVs were identified using CD326-Alexa Fluor^®^ 647 (Alexa Fluor^®^ 647, eBioscience). Nonactivated and activated endothelial-derived EVs were identified using CD146-PC5.5 (cyanine 5.5, Beckman Coulter) and CD62E-PE (phycoerythin, Beckman Coulter) respectively. The incubation of samples with the appropriate isotype controls was subtracted from the positive antibody sample to avoid nonspecific signals. s, calcein-AM (Sigma-Aldrich) was used [26]. True EV events were defined as double-positive stained for: calcein-AM and anti-CD14 (macrophage-EVs); calcein-AM and anti-326 (epithelial-EVs); calcein-AM and anti-CD146 (endothelial-EVs); calcein-AM and CD62E (activated endothelial-EVs).

EV absolute count was expressed as events per microliters of the volume measured by the CytoFLEX. Files were exported, and data were evaluated by CytExpert Software (Version 2.3, Beckman Coulter).

### 4.4. Statistics

Data are shown as means ± SE or median (range). Shapiro-Wilk normality test was applied to evaluate normal distribution of the data. Once it was verified that the data were not normally distributed, nonparametric statistical tests were performed. Group differences were evaluated by Kruskall-Wallis test and Mann-Whitney U test. Correlation coefficients were calculated by the Spearman Rank method. All data were analyzed by R statistical software (version 3.5.2). *p* < 0.05 was considered statistically significant.

## 5. Conclusions

In BAL, significant differences in epithelial and macrophage-derived EVs can be clearly detected between nonsmokers, smokers without COPD, and smokers with COPD, while these differences were not found in plasma. This suggests that BAL could be a better medium than blood to study EVs in lung diseases.

## Figures and Tables

**Table 1:** Clinical characteristics of the subjects in the study.

	Smokers with COPD (n = 9)	Smokers *w*/*o* COPD (n = 11)	Non-Smokers (n = 9)
**Age, yrs**	70 ± 6 *	68 ± 9 *	56 ± 11
**Males/Female n°/n°**	6/3	6/5	6/3
**Smoking history, pack-yrs**	35 ± 9	30 ± 6	
**Current/Ex-smokers n°/n°**	3/6	5/6	
**FEV_1_% pred**	59 ± 3 ^†^	93 ± 8	101 ± 4
**FEV_1_/FVC %**	56 ± 3 ^†^	84 ± 6	89 ± 3
**DLco% pred**	67 ± 11 ^†^	85 ± 4	86 ± 3
**GOLD Stage 1**	3/9	-	-
**GOLD Stage 2**	3/9	-	-
**GOLD Stage 3–4**	3/9	-	-

Values are expressed as mean ± SD. * Significantly different from non-smokers (*p* < 0.05) ^†^ Significantly different from smokers without COPD and non-smokers (*p* < 0.05).

## Data Availability

Not applicable.
